# Supplementary material for: Synergistic effect between plant extracts and fluoride to protect against enamel erosion: An in vitro study
Source: PLoS One. 2022 Nov 16;17(11):e0277552. doi: 10.1371/journal.pone.0277552 (PMC9668169; doi:10.1371/journal.pone.0277552)
Supplement: Attachment — Submitted filename: Reply_to_Reviewers.docx [file pone.0277552.s002.docx]

We would like to thank the reviewers for the suggestions. We have made all the necessary changes and, below, we reply point-by-point to each suggestion.

Reviewer 1:

**This is a welcome addition to the are of erosion research. The study is sound; however, I do have quite a few comments as outlined below.
I am not a fan of ‘absolute’ manuscript titles, esp. since this was ‘just’ an in vitro study. Hence, please either rephrase it or add ‘in vitro’ to the title.**

REPLY: We would like to thank the reviewer for the detailed look into our paper. We agree with your comment to the title and we have changed it accordingly, not only lessening the “absolute” statement, but also adding in vitro, as suggested. It now reads “Synergistic effect between plant extracts and fluoride to protect against enamel erosion: an in vitro study”

**The entire manuscript would benefit from some editing. Here are just a few examples from the abstract.
Line:
23 – ‘such as’ rather than “like”; comma after “teas”
24 – ‘explored’ not “explore” (past tense throughout)
25-26 – It would be better to write ‘We distributed enamel specimens into 9 groups…”
26 – ‘F-‘ not “F-“ (entire manuscript actually; superscript “-“, this may be lost as a result of the Editorial Manager); the same applies to Sn2+ (31)
32 – ‘specimens’ not “speicmens”
32-24 – The description of the experimental procedure could be expanded. Were the cycles performed continuously without any prolonged remineralization phases after each cycle? Was it all done within one day? Even the description in the m&m section is unclear. The way it is written would suggest it was all done in one day. What is the relevance of this procedure? This would relate to five (fluoride) treatments within a roughly 8-hour period.
35 – ‘into’ not ‘to’
38 – ‘For’ not “In”**

REPLY: We have corrected all the typos. We also added more information to the m&m section.

**38-40 – I do not think there is a need to state that both factors affected rSH when their interaction was significant. To make it easier for the reader, perhaps the authors can add some actual rSH data? I would also suggest to leave the rSRI and calcium data to the main text and focus more on the primary outcome measure rSH, assuming it was.**

REPLY: We understand your point of view, but since we still had some room left with the number of words, we decided to still give the readers the overview of these results in the abstract.

**45-46 – I do not think the authors can derive this conclusion from their study as the extracts were not tested in a model without salivary proteins.
48 – perhaps ‘most promising’ rather than “best”. Or perhaps leave this out entirely: “yielding the best results”.
45-48 – I would suggest something along these lines to be a more truthful conclusion: ‘We conclude that polyphenols from plant extracts, when combined with fluoride, improve the protection against enamel erosion in a model with salivary proteins.’**

REPLY: We agree with all the above points related to the conclusion, so we have changed it here and in the body of the manuscript. The main sentence now reads: “We conclude that polyphenols from plant extracts, when combined with fluoride, improve the protective effect of salivary pellicles against enamel erosion”

**I would suggest moving the “experimental design” section to the front of the m&m section.**

REPLY: We have moved the experimental design part, as suggested.

**Why were the acid solutions pooled rather than analyzed individually?
How reliable are the calcium data as the treatments could have modified the pellicle layer to attract more or less calcium from the saliva? In other words, how much of the calcium that was solubilized was from the specimens vs. the pellicle?**

REPLY: The reviewer asks very pertinent questions. In previous studies, we used to analyse the calcium results individually, for each cycle. These results were always quite linear, and they made more sense when presented as cumulative results – in other words, at the end of the study we had the total amount of calcium released. These were the values used for the statistics. By pooling the aliquots, we already get this final “cumulative/total” result, so the only difference is less experimental steps (and thus less room for biases). But the reviewer is correct in that the calcium can be released from the pellicle and/or the specimen. From the present methodology, we cannot separate which calcium ions came from which part. To answer such question, we would need a totally different methodological approach, with a completely different objective. This is a very interesting point, and we will investigate this question in a future study.

**Statistical analysis: While I would consider myself anything but an expert, I do not understand why the authors removed specimens from groups without outliers for the sole purpose to have balanced groups. This does not make sense to me. Just consider how clinical trials are being conducted. No subject would be excluded based on such a criterion. I would also like to see more detail about the procedure to test for outliers.**

REPLY: We understand the reviewer’s concern. However, this was necessary to perform parametric tests.

From the normality tests for SH and SRI, we observed that some groups did not have a normal distribution, mostly because of 1 outlier. Once we removed this 1 outlier from these groups, the latter presented a normal distribution. This would then allow for parametric tests. However, parametric tests are fairly robust when sample sizes are equal, whereas when sample sizes are not equal, the F-statistic is affected by the skew, decreasing the power of the analyses. Therefore, in order to be able to perform more robust parametric tests, we had to make the sample sizes equal – in other words, delete 1 specimen from each of the groups. If the groups already presented normal distribution, the 1 value was chosen at random (by generating random numbers). In the end, each group had equal number of specimens (n=14), they all presented normal distributions, and we were able to perform GLM. For the calcium results, however, even with 14 specimens the groups did not pass the normality test. So, for calcium, we performed non-parametric tests.

To give you more details related to the testing of the outliers, we identified them with boxplots, as in the example below:

These Boxplots were made with SPSS, following Tukey’s definition. According to Tukey, the middle line corresponds to the median, the upper (H1) and lower (H2) limits of the boxes are the values that lie midway between the median and the extreme values (25^th^ and 75^th^ percentiles, respectively), and the whiskers extend 1.5 times the length of the box (i.e. 1.5*(H2-H1) but stopping at the last value in the data set previous to this calculated value. So, whatever lies beyond are the outliers.

**Results: A significant interaction between study factors does not necessarily mean what the authors stated in lines 230-232. It simply means that they interact; it could well have been that one extract enhanced the protection afforded by fluoride when another did the opposite.**

REPLY: We agree with the comment, and we deleted the ambiguous phrase.

**Now that I have seen figure 1, I cannot see how the authors can justify their conclusion in the abstract (“We conclude that polyphenols from plant extracts seem to interact with salivary proteins, modifying the pellicle and improving the protection against enamel erosion,…”). None of the extracts were different from deionized water in the absence of fluoride.**

REPLY: We also agree. We changed the sentences in the abstract and manuscript. T now reads: “We conclude that polyphenols from plant extracts, when combined with fluoride, improve the protective effect of salivary pellicles against enamel erosion”

**The authors argued against the usefulness of both rSRI and calcium in the discussion and questioned their relevance. Why did the authors include these data anyway, let alone decided to include these methods during project development?**

REPLY: This a very fair question, but these methods also reveal important information. First, we have carried out most of our studies on pellicle and plant extracts with these methods, so it is highly recommendable to keep including these results for comparison reasons. Second, these methods bring important information related to the pellicle. SRI correlated with roughness, and calcium reveals how much tooth mineral is being demineralized. Even if we cannot separate which calcium ions were released from the pellicle or from the tooth, we can still clearly see that the non-modified pellicle released more calcium (so it protected less). So, it is recommended to keep these methods in this study.

**The authors’ attempt to rationalize their findings is interesting. They could have investigated calcium release from ‘extract’ treated specimens, previously exposed to saliva or not, under neutral or basic conditions (e.g., wash specimens with NaOCl and analyze the NaOCl solutions for calcium in a model without acid challenges?) to support at least one of their theories.**

REPLY: This is a very good point. We will indeed investigate this and design a future study specifically related to your suggestion. We are confident that such a study may shed some light on the mechanism of action of the extracts. Thank you for the suggestion.

**The last paragraph is somewhat negating a possible manuscript. It is highly unusual to do what the authors did and reminds me somewhat of a TV series… Perhaps the authors would be better off highlighting the limitations of their present study to help the reader putting this all into context?**

REPLY: We agree with the suggestion, and we have completely reformulated this last paragraph. It now reads:

“Remarkably, the synergistic effect between fluoride and polyphenols yielded even better protection than the fluoride-only or the positive control (stannous-containing) solutions. These results, however, should still be viewed with the caveat that this study has some limitations. Primarily, even though we used human saliva, we formed the salivary pellicle *in vitro*, which can be considerably different from those formed *in vivo* [44]. Secondly, although we used human teeth, the specimens were ground to reach a healthy (non-demineralized) layer of enamel. But teeth that already present signs of erosive tooth wear may have different susceptibilities to further demineralization [45, 46]. Thirdly, the present study used an initial erosion model, where the demineralization is limited to the surface of the specimens and no bulk surface loss has occurred. Future studies should consider how the modified pellicle would perform under such severe conditions and also under abrasive impacts.”

**Reviewer #2: The text is well written and the results are supported by the methodology performed. The study of plant extract as potential preventive and protective tools against erosive wear has shown interesting and promissing results by recent literature, and this study corroborates with it. However the results presented are very preliminary, and although human saliva was used, the in vitro results showed only the protective potential in initial erosion, without considering a more severe erosive cycling. Also, abrasion was not considered in the text, and shall be listened as an limitation of the work.**

REPLY: Thank you very much for your kind review. Indeed, these are limitations to the study, and we have now addressed them. Reviewer 1 asked to reformulate the last paragraph, and we addressed these issues there. It now reads:

“Remarkably, the synergistic effect between fluoride and polyphenols yielded even better protection than the fluoride-only or the positive control (stannous-containing) solutions. These results, however, should still be viewed with the caveat that this study has some limitations. Primarily, even though we used human saliva, we formed the salivary pellicle *in vitro*, which can be considerably different from those formed *in vivo* [44]. Secondly, although we used human teeth, the specimens were ground to reach a healthy (non-demineralized) layer of enamel. But teeth that already present signs of erosive tooth wear may have different susceptibilities to further demineralization [45, 46]. Thirdly, the present study used an initial erosion model, where the demineralization is limited to the surface of the specimens and no bulk surface loss has occurred. Future studies should consider how the modified pellicle would perform under such severe conditions and also under abrasive impacts.”

**Reviewer #3: Title: There is a synergistic effect between plant extracts and fluoride to protect against enamel erosion
This is an interesting study regarding the synergistic effect between plant extracts and fluoride against enamel erosion. Here are some points that should be addressed:**

REPLY: Thank you for your review. We have made all the suggested changes.

**- The surface hardness measurements only give information regarding the surface, the softening characteristics of the surface, but not the amount of tissue that was lost, which can be evaluated by measuring surface loss using for example optical profilometry. So, surface hardness can only offer qualitative information for the nature of the erosive challenge.**

REPLY: Yes, the reviewer is correct in that surface hardness only gives us an idea of the characteristic of the surface. However, in this mild model we used, where erosion is in its early stages, there is only a surface demineralization of the tooth tissue. There is no bulk loss (surface loss) yet. We only see some surface loss after a more severe experiment. For this reason we did not use the profilometer. The issue is addressed in the last paragraph, in the sentence “…the present study used an initial erosion model, where the demineralization is limited to the surface of the specimens and no bulk surface loss has occurred…”

**- It should be more informative to provide images of the surface of the specimens after the erosive challenge to evaluate also qualitatively the erosive activity on each experimental group.**

REPLY: The Reviewer brings up an interesting question, one that we have also already asked previously. In an earlier study, we also used different polyphenol-rich solutions and we took some SEM images of the specimens, to verify how the enamel surfaces looked like. Even though we were able to measure a significant difference in the protective effect of pellicle modification between the groups, these differences were not so clearly observed in the images. Below we offer you an example of an experimental group (pellicle modification with a polyphenol-rich solution) and of a control group (no pellicle modification – where deionized water was used), and we see that the qualitative differences are not so clearly distinguishable as we had hoped. For such reason, adding these images to the publication would, unfortunately, not complement the results. We do thank the Reviewer for the valid and well-thought suggestion.

SEM images of enamel surface (1000x magnification) after the experimental cycles with modified (experimental group) and non-modified (control group) salivary pellicles.

| Experimental group | Control group |
| --- | --- |
|  |  |

**- Preventive measures against erosion are usually suggested when there is early diagnosis of erosive tooth lesions and not in intact teeth. Moreover, different enamel lesion depths may have different susceptibility to demineralization. As a result, this should be mentioned as a limitation of this study.
Carvalho TS, Baumann T, Lussi A. Does erosion progress differently on teeth already presenting clinical signs of erosive tooth wear than on sound teeth? An in vitro pilot trial. BMC Oral Health 2017;17:14.**

REPLY: Yes, the Reviewer is correct, and this is a very important point. This is a limitation of our study. Reviewer 1 had also suggested to reformulate the last paragraph, so we included this information there. It now reads:

“Remarkably, the synergistic effect between fluoride and polyphenols yielded even better protection than the fluoride-only or the positive control (stannous-containing) solutions. These results, however, should still be viewed with the caveat that this study has some limitations. Primarily, even though we used human saliva, we formed the salivary pellicle *in vitro*, which can be considerably different from those formed *in vivo* [44]. Secondly, although we used human teeth, the specimens were ground to reach a healthy (non-demineralized) layer of enamel. But teeth that already present signs of erosive tooth wear may have different susceptibilities to further demineralization [45, 46]. Thirdly, the present study used an initial erosion model, where the demineralization is limited to the surface of the specimens and no bulk surface loss has occurred. Future studies should consider how the modified pellicle would perform under such severe conditions and also under abrasive impacts.”
